# In Vitro Dissolution of Na-Ca-P-Oxynitrides

**DOI:** 10.3390/ma14237425

**Published:** 2021-12-03

**Authors:** Natalia Anna Wójcik, Polina Sinitsyna, Sharafat Ali, Leena Hupa, Bo Jonson

**Affiliations:** 1 Advanced Materials Center, Institute of Nanotechnology and Materials Engineering, Gdańsk University of Technology, 11/12 G. Narutowicza Street, 80-233 Gdańsk, Poland; 2 Department of Built Environment and Energy Technology, Linnaeus University, 35195 Växjö, Sweden; sharafat.ali@lnu.se (S.A.); bo.jonson@lnu.se (B.J.); 3 Johan Gadolin Process Chemistry Centre, Åbo Akademi University, Piispankatu 8, 20500 Turku, Finland; polina.sinitsyna@abo.fi (P.S.); leena.hupa@abo.fi (L.H.)

**Keywords:** bioactive glass-ceramics, simulated body fluid, oxynitride glass-ceramic, in vitro dissolution

## Abstract

Sodium-calcium-phosphate based oxynitride glasses and glass-ceramics doped with Mg, Si, and Nb were studied in vitro in simulated body fluid (SBF) under static conditions. The release of ions and pH changes up to 7 days of immersion were investigated. The nitrogen incorporation into phosphate glass matrix was found to notably influence in vitro dissolution only of homogenous glasses. Increasing the nitrogen content in the samples decreased the mean mass loss, while the niobate incorporation increased it. The correlation between the nitrogen content and increase in pH of SBF was also observed. The presence of phosphates crystallites was found to support the dissolution process at the beginning step (up to 3 days).

## 1. Introduction

Congenital abnormalities, traumatic injury, and/or disease might damage the human bone. Therefore, bone is one of the most commonly transplanted tissues [1]. Synthetic materials such as bioactive glasses and glass-ceramic composites are frequently studied as implant materials for bone replacement [2,3,4,5,6]. These materials can chemically bond with the bone and potentially enhance new bone growth used to replace damaged bone. Silicate- and phosphate-based bioactive glasses are well-known non-toxic and highly biocompatible bone graft materials [7,8,9,10]. When exposed to body fluids, an amorphous calcium phosphate layer forms at the glass surface. Subsequently, this layer crystallizes into bio-mimetic hydroxyapatite (HAp, Ca_10_(PO_4_)_6_(OH)_2_) that provides a bone-glass interface [11]. However, besides the changes that occurred on the glass surface, the changes that proceeded in the solution should be studied to fully describe the dissolution of bioactive glasses. Usually, the in vitro dissolution experiments are carried out in aqueous solutions (simulated body fluid–SBF, phosphate buffered solution–PBS and Tris-buffered solution) under static conditions, which allows to analyze the pH changes during immersion [12,13,14]. Moreover, the ion release study of the solution gives information on how different glasses behave in the initial stages of the immersion and also shed light on the bioactivity of the glasses [15].

Recently, calcium-phosphate glasses with their chemical composition close to natural bone have been intensively studied due to their fast dissolution considered favorable for bioactive materials [1,16,17,18]. The CaO content decreased the dissolution rate of phosphate glasses in distilled water and simulated body fluid (SBF) due to the improvement of the phosphate network. Furthermore, the cytotoxicity of the Na-Ca-P-O glass system also decreased with an increase in the CaO and a decrease in the P_2_O_5_ content [19,20,21,22]. The addition of Na_2_O to the phosphate glasses controls their solubility degree in physiological fluids [23].

In contrast, a too high aqueous dissolution of phosphate glasses can be a limitation for their application. The dissolution rate can be influenced by improving the chemical durability through increased network connectivity induced by nitrogen incorporation [24,25,26]. Niobium addition can also significantly improve the thermal stability and chemical durability of the phosphate glasses and glass-ceramics [24,27]. Moreover, niobium has been shown to stimulate mineralization in human osteoblast populations [28]. Furthermore, doping phosphate glasses with MgO is also known to reduce the biodegradation rate of Na-Ca-P-O glasses [29] and slow down the rate of formation of the hydroxyapatite layer on their surface [30].

In our previous work [24], we have synthesized Na-Ca-P-O glasses and glass-ceramic composites doped with Mg and/or Si_3_N_4_. The results suggested that nitrogen was incorporated in the network of the homogeneous glasses and glass-ceramics. The glass-ceramics had high niobium content (up to 1.8 at%), and they consisted of calcium and sodium phosphate nanocrystallites and hydroxyapatite (HAp). The presence of HAp implied that these glass-ceramics might possess bioactive properties [10,31,32] and thus be alternatives to the well-known bioactive phosphate glasses. Accordingly, this work aimed to explore the in vitro dissolution of HAp-containing oxynitride glasses and glass-ceramics.

## 2. Materials and Methods

### 2.1. Tested Materials

In total, 12 samples with different compositions were synthesized: 5 in the air (series I) and 7 in nitrogen (series II and III) [24]. The samples were prepared through a two-step synthesis route. In the first step, the matrix glass 40Na_2_O-20CaO-40P_2_O_5_ (in mol%) was prepared by the conventional melt quenching technique (glass ID g0 in Table 1). Batches of about 10 g were melted from the reagents NaH_2_PO_4_ (≥99.5% SIGMA ALDRICH), Na_2_CO_3_ (99.9 + % ChemPur GmbH, Karlsruhe, Germany) and CaCO_3_ (99.9 + % ChemPur GmbH) in porcelain crucibles at 850–950 °C in air for 30–60 min.

In the second synthesis step, an appropriate amount of the powdered matrix glass g0 was re-melted together with different amounts of Si_3_N_4_ (99.99% ChemPur GmbH) and Mg (99.8% ABCR GmbH&Co, Karlsruhe, Germany). The samples in series I were doped with 0.5, 1, and 2 mol% Si_3_N_4_: (glasses IDs: g2, g3, and g4, respectively). They were melted in similar conditions to the matrix glass. Additionally, a reference glass doped with 1 mol% of SiO_2_ (99.99% ChemPur GmbH) was prepared (glass g1). All samples were annealed at 350 ^°^C for five hours and then cooled for 10 h to room temperature.

In series II, the matrix glass (glass g0) was re-melted with 1 mol% of Mg and 1, 2, and 3 mol% of Si_3_N_4_ (IDs: g5, gc6, and gc7). Series III samples were prepared from pre-prepared 1Si_3_N_4_-99(40Na_2_O-20CaO-40P_2_O_5_) (glass g3) and 2, 3, 4, and 5 mol% of Mg metal (IDs: g8, gc9, gc10, and gc11). Batches of about one gram of each composition were melted in niobium crucibles in the radio-frequency furnace under a nitrogen atmosphere for one hour at 1200–1550 °C, depending on the composition. The melts were cooled by turning off the furnace at the end of the run, giving an approximate cooling time of one hour to room temperature.

The compositions of all samples were examined with a high-resolution scanning electron microscope (LYRA3, TESCAN, Brno, Czech Republic) equipped with EDX detector (EDX; energy dispersive X-ray spectrometer) on samples surfaces coated by~40 nm carbon. Each composition was checked three times. An additional information about the measurements of the chemical composition as well as the structure and thermal properties of these glasses and glass-ceramics is reported in [24].

### 2.2. In Vitro Bioactivity Measurements

In vitro dissolution experiments were carried out in simulated body fluid (SBF) under static conditions. The solution was prepared according to the protocol given by Kokubo et al. [19] For static dissolution studies, 100 mg of single pieces samples were added in 4 mL of SBF and placed in an incubating orbital shaker (at ambient atmosphere) held at 37 °C and agitated at 100 rpm. After 3 and 7 days, the samples were removed from the solutions, dried at 37 °C and weighed. The total percentage change in weight was calculated based on the mass before and after the dissolution. As the sample preparation procedure was laborious, the in vitro measurements were performed parallel for two pieces of each sample for two time points, i.e., 3 and 7 days.

The solution pH was measured at several immersion times to provide information on the pH trends and understand the dissolution behavior. Moreover, several consecutive pH measurement points were applied to check the differences between every consecutive measurement. Calibration of the pH meter (Mettler Toledo SevenEasy^TM^ S20, Zurich, Switzerland) was carried out using three different pH standards (pH 4.01, 7 and 9.21). The main observed discrepancy between the two consecutive measurements was 0.05 pH units. After 3 and 7 days, the supernatant was separated from the samples by filtration and analyzed with an inductively coupled plasma optical emissions spectrometer (ICP-OES, PerkinElmer Optima 5300 DV, Shelton, CT, USA). The particles were immersed in resin after which their cross-sectional areas were revealed by cutting and polishing. The surfaces of the samples were studied using SEM-EDX (Leo 1530; Leo, Oberkochen, Germany) and compared with analyses of the sample surfaces before the immersion.

## 3. Results

### 3.1. Mass Change during the Dissolution in SBF and SEM Observations

In vitro dissolution tests in SBF were conducted for 12 samples. The compositions of the as-prepared samples were reported in our previous paper [24] and are included in Table 1. The label g before the sample number referred an amorphous sample, while gc referred a glass-ceramic sample. Most of the samples contain trace amounts of Al (0.1–0.3 at %) from the ceramic crucible. However, the Al content in samples gc10 and gc11 was markedly higher (≥3.7 at %). Additionally, quite a high amount of K (up to 4.7 at %), originating from the crucible coating, was measured in samples. Samples in series II and III also contain niobate (0.3–1.8 at %), dissolved from the crucible during the second melting. A similar dissolution of niobate in oxynitride silicate-based glass melts has not been observed previously [33,34,35,36,37]. The morphology and the structure of all samples before immersion have been reported in our previous paper [24]. All glasses were partially transparent, mostly homogeneous and amorphous, while all glass-ceramic samples were translucent and contained crystallites of calcium and sodium phosphates, including hydroxyapatite. The approximate content of crystalline phase increase for samples in order gc6 < gc7 < gc9 < gc10 < gc11. The XRD patterns of glass-ceramic samples are presented in Figure S1 (taken from [24]). The mass loss of samples was measured after initial stage (3 days) and long term (7 days) of dissolution in SBF. Table 2 shows the % mass changes and mean % mass changes calculated per day, to simplify the comparison. The values show differences in the mass changes of the samples during the SBF immersion. The matrix glass had lost 5% of its initial mass after 3 days of immersion. The glass continued to dissolve (mass loss up to 7%) over the following 4 days at a slower rate. Reference glass g1 doped with a small amount of Si (0.4 at%) showed an opposite trend. The mass of sample g1 increased during the first 3 days, while during the next 4 days, the glass started to dissolve, as indicated by the 1% loss compared to the initial mass. Series I samples were doped with different contents of Si and N. Glass g2 contains 0.8 at% of N and slightly less Si, 0.2 at%, compared to the reference glass g1. The sample had dissolved mainly during the first 3 days of immersion, as suggested by mass loss measurement (13%). Glass g3 contains more Si (1.2 at%) than g2. Glass g3 dissolved continuously in SBF during the 7 days, similarly to the matrix glass g0. However, the mean % mass loss calculated per day was higher for g3 compared to g0. During the first 3 days of immersion, the mean % mass loss of g3 was 2.67% per day, while during the long-term immersion, it was 3.29% per day. However, glass g4, which contains 1.3 at% of Si and 2.1 at% of N showed a similar dissolution trend to glass g1. At the beginning of the SBF immersion, its mass increased by 7% during the first 3 days, after which its mass slightly decreased but was still 5% higher than the initial mass on day 7. Series II and III showed different behavior compared to the series I and reference glasses. After the first 3 days, the mass of series II and III samples showed a lower mass than before the immersion. The lowest mass loss, 9%, was measured for gc6 while the highest mass loss was 25% for gc7. During the next 4 days of immersion, the mass of series II and III increased. After 7 days, the total mass loss varied between 2% for glass g8 and 12% for gc7.

**Figure 1:**
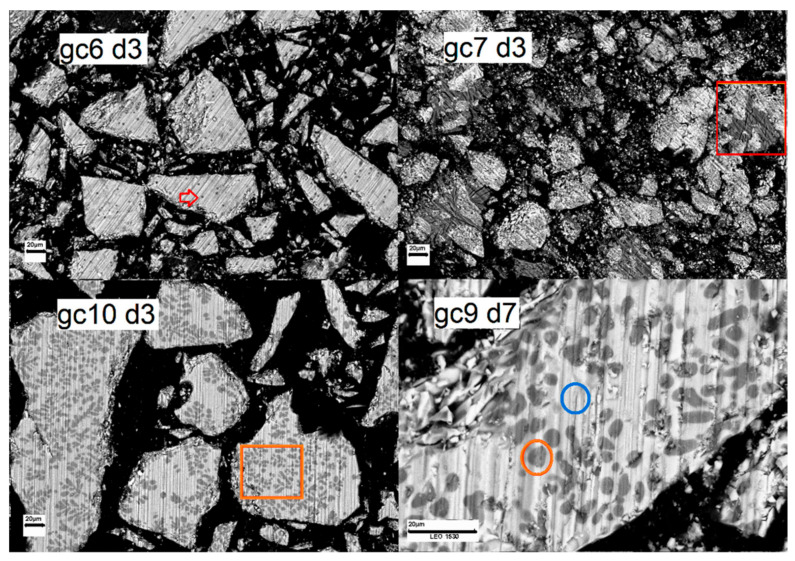
SEM (secondary electron) images of exemplar samples: gc6 (**up left**), gc7 (**up right**), gc10 (**down left**), and gc9 (**down right**) after immersion in SBF for 3 or 7 days. The scale bar, 20 μm, reveals the magnification of the images.

SEM micrographs of the cross-sectional surfaces after immersion in SBF for 3 and 7 days are given in Figures: (Figure S2) reference glasses g0 and g1, (Figure S3) series I glasses g2, g3, and g4, (Figure S4) series II samples g5, gc6, and gc7, and (Figure S5) series III samples g8, gc9, gc10, and gc11, respectively. The marks: d3 and d7 indicate 3 days and 7 days of immersion, respectively. The scale bar shows a value 20 μm. The topography of all samples was not homogenous after immersion for 3 and 7 days in SBF Samples exhibit inhomogeneities (visible as brighter structures) dispersed unevenly on the particle outer surfaces. Most samples additionally contain inhomogeneities visible as darker points, stains, or cauliflower-like structures placed on brighter structures which were also found before immersion in SBF [24]. Figure 1 presents exemplar samples with marked inhomogeneities: (up left) gc6, (up right) gc7, (down left) gc10 and (down right) gc9. The darker points are especially observed for samples: g5 and gc6 (indicated by the red arrow in Figure 1 up left). Sample gc7 contains cauliflower-like structures, exemplary marked by the red frame in Figure 1 up right. The stains are clearly visible for samples: gc 9, gc10, and gc11 (marked by orange frame and circle in Figure 1 down left and right).

Table S1 shows the changes in the chemical composition of the samples surfaces after immersion in SBF for 3 and 7 days. These changes were evaluated using SEM-EDX analysis (exemplar results showed in Figure S6). Table S1 presents also the chemical composition of samples before immersion in SBF as mentioned before. According to the results, the composition of all samples changed after immersion in SBF. Major changes were observed for the oxynitride samples. Starting with the target glass g0, the cross-section composition only slightly changed: the K and Ca contents decreased while Na quantity increased after immersion in SBF. Unlike to g0 the major and mostly contradictory changes were found for reference glass g1 and for all other samples. It is worth underlining that the chemical composition of cross-section obtained for samples after 3 and 7 days in SBF was mostly congruent and the biggest changes took place in the first 3 days. Therefore, for part of glasses (g2, g4 and g5) EDX measurement was conducted only after 7 days in SBF. Comparing results of glass g1 and all series I, II, and III samples before and after the biodegradation test, it may be seen that in all of them the content of P noticeably decreased as well as quantities of Na and Mg (in series II and III samples). For most samples, the quantity of Si after immersion stayed analogous to the ones before. Interestingly, the content of Ca was found to be similar after static dissolution for samples: g5, gc6, lower for samples: g1, g2, g3, g4, g8, gc10, and gc11 or significantly higher for sample gc7, than before. For sample gc9 the content of Ca increased after 3 days in SBF and decreased after 7 days in SBF while comparing with value before immersion. The Nb amount (in series II and III samples) was found to be higher than before immersion. Similarly, to Nb also Al content increased after the SBF test for most of the samples and for the others (series II samples and gc10, gc11) it stayed comparable to values before immersion.

The orange point in Figure 1 indicates the place of inhomogeneity, while the blue one represents the surrounding area. The EDX analysis showed that the composition of visible darker stains contains higher amounts of Na, P, and Ca compared to the brighter area (showed in Figure S6). Similar results were found also for cauliflower-like structures. Moreover, there was a lack of Si and Nb, while the content of Al and K is only traced in stains/cauliflower-like structures. The calculated molar ratio Ca/P for stains/cauliflower-like structures is approximately 0.75. This value is two times higher than the one obtained for overall compositions ~0.30 (see Table 1) while the theoretical Ca/P-ratio in hydroxyapatite (Ca_10_(PO_4_)_6_(OH)_2_) is 1.67. However, the size of visible structures was too low to confirm their exact compositions by EDX measurements, which showed also the results of matrix.

The SEM-EDX measurements are helpful to show the overall picture of composi-tional changes [38,39,40] however, they examined chemical composition only of selected areas of samples. Therefore, SEM-EDX results may have big error margins and due to this fact, the discussion part mostly focused on ICP-OES results.

### 3.2. Ion Concentrations and pH of SBF

**Figure 2:**
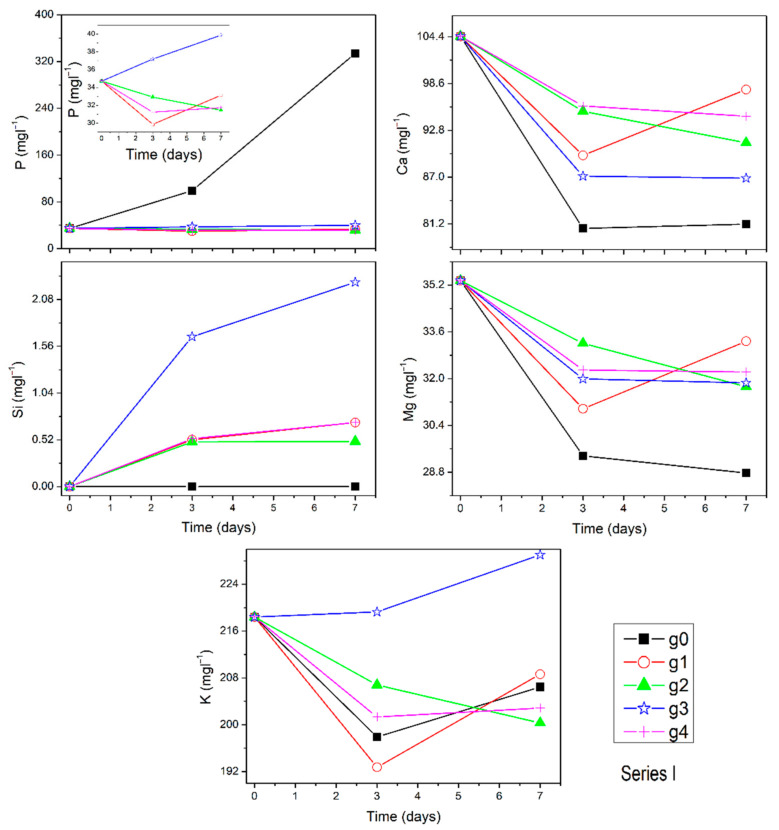
Ion dissolution profiles of series I samples in SBF under static conditions as function of immersion time. Error bars are included in the points’ size.

**Figure 3:**
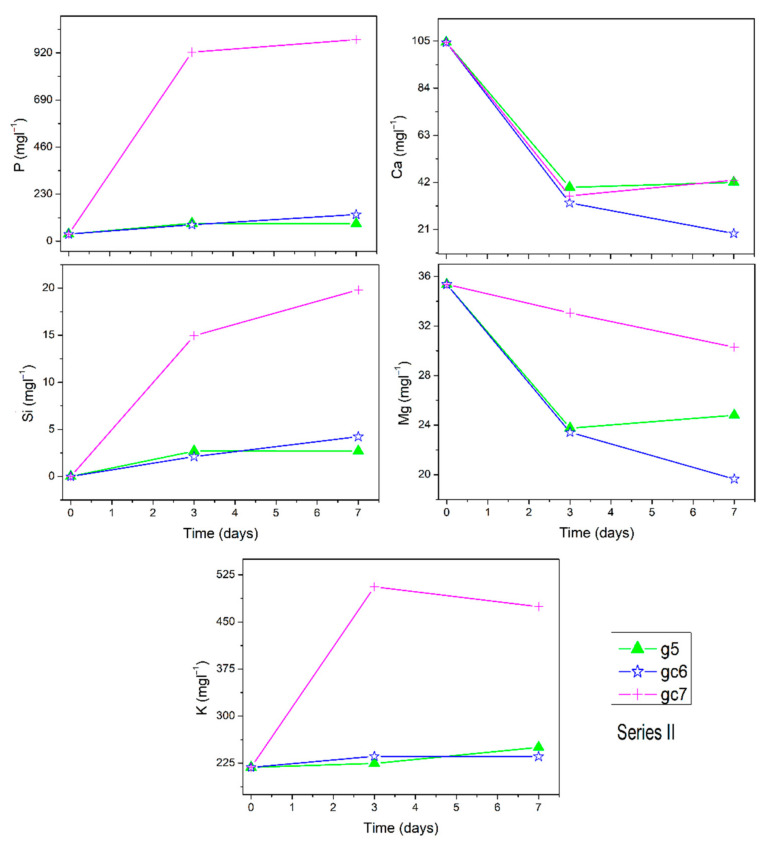
Ion dissolution profiles of series II samples in SBF under static conditions as function of immersion time. Error bars are included in the points’ size.

**Figure 4:**
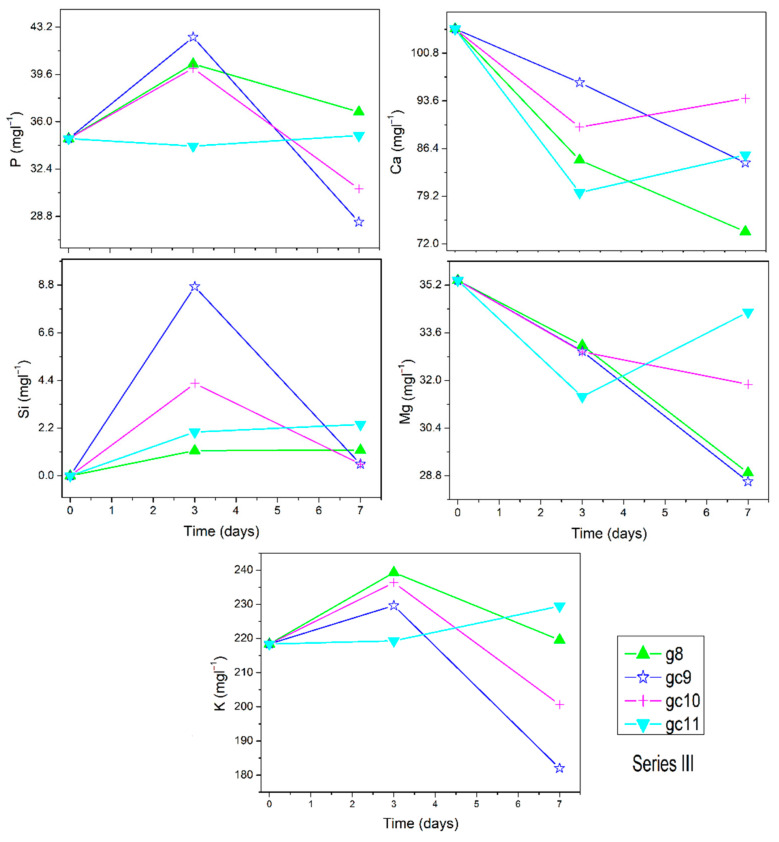
Ion dissolution profiles of series III samples in SBF under static conditions as function of immersion time. Error bars are included in the points’ size.

The ion dissolution profiles of Si, Ca, K, P, and Mg for series I, series II, and series III are presented in Figure 2, Figure 3 and Figure 4, respectively. Insert in selected Figure 2 shows the concentrations in bigger magnification for comparison. For the matrix g0, reference g1, and series I glasses, it can be seen that the dissolution profile for P ion shows reliable trends for all samples. P ion concentration in SBF increases for g0 (from 35 up to 334 mgl^−1^) but stays constant for other samples. The dissolution profiles of Ca ion show clear decreasing trends for all samples except g1 at the longest time point (7 days). Si dissolves from Si_3_N_4_ doped samples and reference glass g1. The dissolution profiles of K ion concentration decrease but not for glass g3, analogously for Mg ion with the exception of reference g1. In the series II, P ion concentration in SBF increases for gc6 and gc7 but is constant for g5. Ca ions concentration decreases in SBF for all series II samples. Si and K ions dissolve from all series II samples into the SBF. Mg dissolution profiles decrease for all samples. In series III, P ion concentration in SBF stays almost constant while concentration of Ca, Si, and Mg ions decreases, and K ion dissolution profiles increase. The Na concentration in SBF (not shown here) is very high and for most samples it almost stayed constant, within the accuracy of the measurement. Only for gc7 it was found to increase.

**Figure 5:**
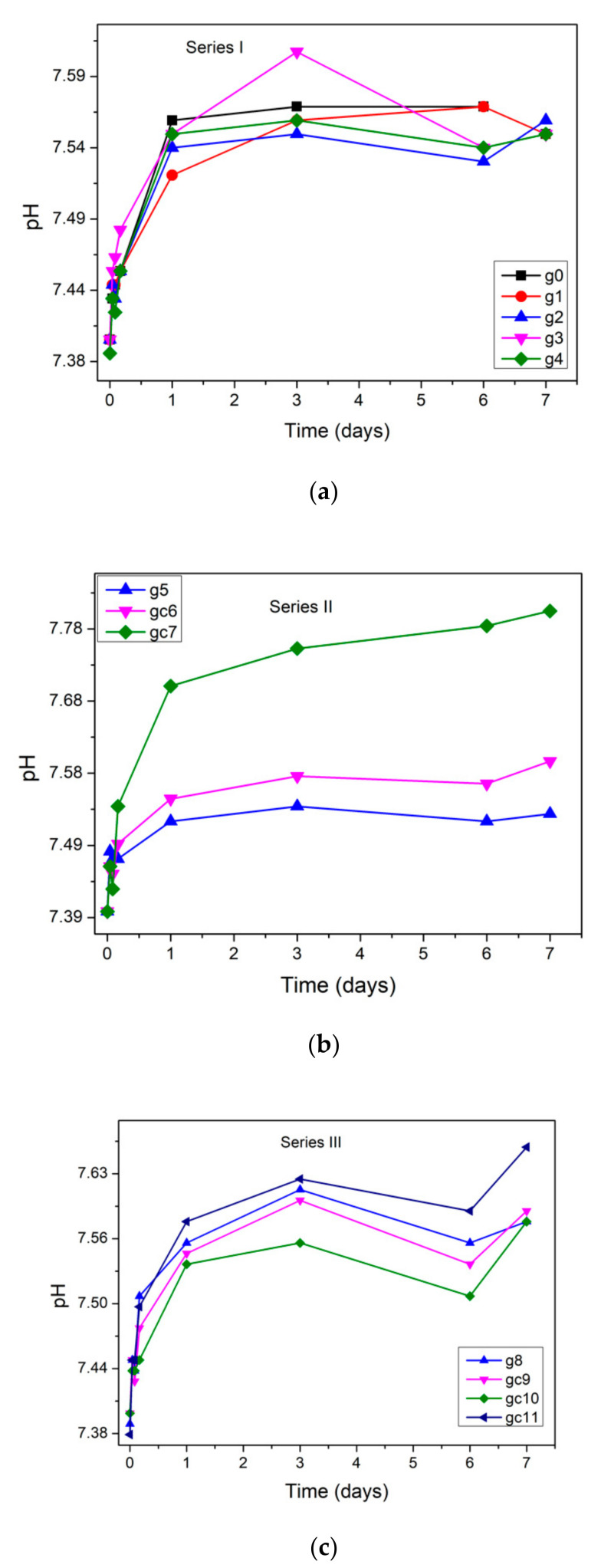
pH variations of solution after immersion of (**a**) series I, (**b**) series II, and (**c**) series III samples in SBF up to 7 days.

Figure 5 shows the pH changes as a function of immersion time in static SBF. It can be seen that the pH of SBF increased from 7.4 to above 7.5 during the first day of immersion for all samples. Afterward for glasses: g0, g2, g4, and g5 it stayed approximately constant. After 3 days of static dissolution, the pH values slightly decreased for glasses g3 (from 7.61 to 7.54) and g8 (from 7.61 to 7.56). Different behavior was observed for the glass-ceramic materials: gc6 and gc7, the pH of SBF increased with time during all dissolution tests while for samples: g3, gc9, gc10, and gc11 it increased for the first 3 days and afterward decreased. The highest pH (7.8) was observed for SBF after immersion of sample gc7 for 7 days.

## 4. Discussion

The dissolution process of the phosphate-based glasses in SBF includes the hydration process in which a hydrated layer is formed on the glass surface based on the ion-exchange reaction of Na^+^ and H^+^ [29]. The dissolution process consists also of the breakage step which indicates the continuous attack of water results in the breaking up of P–O–P bonds and consequently the breaking of glass network and release of [PO_4_]^3−^ units into solution [29]. The phosphorus cations released from the breakage of P–O–P bonds tend to bond with protons to form phosphoric acid and therefore the decrease in pH of SBF (pH 7.4) can be observed after dissolution of phosphate glasses [41]. On the other hand, the release of Na^+^, Ca^2+^, and Mg^2+^ ions into the solution and chelation with the released phosphate species increases the pH of the solution [41]. Therefore, the correlation of pH changes with ion releasing profiles and sample mass loss may indicate the information about the dissolution of glass.

El-Kheshen et al. [42] reported the acceleration effect of low concentration of Al_2_O_3_ (up to 2 wt%) on the formation of HAp layer on the surface of phosphate glass immersed in SBF. However, higher concentrations of Al_2_O_3_ (between 3 and 10 mol%) in phosphate glasses inhibited dissolution of glasses and acidification of PBS solution while the glass containing 10 mol% of Al_2_O_3_ showed the best cytocompatibility and bioactivity results [43]. According to the literature, we may suppose that in most of our samples Al_2_O_3_ (up to 0.5 mol%) has a positive influence on the HAp layer formation while in the case of gc10 and gc11 (Al_2_O_3_ > 6 mol%) it may slow down the dissolution in SBF. Moreover, doping phosphate glass with Al_2_O_3_ improves its long-term stability, which is important for using bioactive glass as bone implants [44].

The matrix glass g0 was found to dissolve continuously in SBF for up to 7 days. The observed decrease in mass was due to the high P release into SBF, which had the highest point (up to 334 mgl^−1^) during the last 4 days of immersion. At the same time, the highest ion release (Ca^2+^, K^+^, Mg^2+^) from SBF was observed during the first day of immersion as visible in pH increase (up to 7.56). The increase in pH values suggests typical dissolution behavior under static conditions [13,14] and the possible chelating of released phosphate species with Mg^2+^ and Ca^2+^ ions present in SBF. Doping with a small content of Si had a strong influence on the solubility properties of g0 glass. An increase in mass (negative value of mass loss in Table 2) was observed for glass g1 during the first 3 days of immersion in SBF. A similar trend was also indicated for poly(ε-caprolactone)-bioactive glass composites [45] and poly(lactide-*co*-glycolide) scaffolds [46] and it was related to the HAp layer formation on the material surface. During the next 4 days of immersion, glass g1 decreased its mass as a result of few possible processes: dissolving itself and/or dissolving residual H_2_O into SBF [45]. In the case of g1 glass, the possible transfer of Ca^2+^ from the SBF to the glass surface can explain the increase in mass with time while dissolving of Si^4+^ can be responsible for the decrease in mass. Series I glasses after the incorporation of small amounts of Si and N were still found to be amorphous and homogenous before immersion [24]. For all of them, the ion release profiles and the pH changes of SBF suggest that transfer of Ca^2+^, K^+^, and Mg^2+^ from SBF onto the material surface and release of Si during the dissolution in SBF occurred as one of the first events after 1 day of immersion. The Si^4+^ behavior indicated that the Si-O (-P) bond is weaker than Al-O (-P) in these glasses and breaks in contact with SBF [47,48]. All samples of series II and III lost the highest % of their mass during the first 3 days of immersion due to the release of Si^4+^ and K^+^ into SBF and for gc6 and gc7 also P^5+^. The highest mass loss was observed for gc7, as a consequence of releasing the highest amount of P^5+^ into SBF. Exceptionally this sample also showed the release of Na^+^ into the SBF.

**Figure 6:**
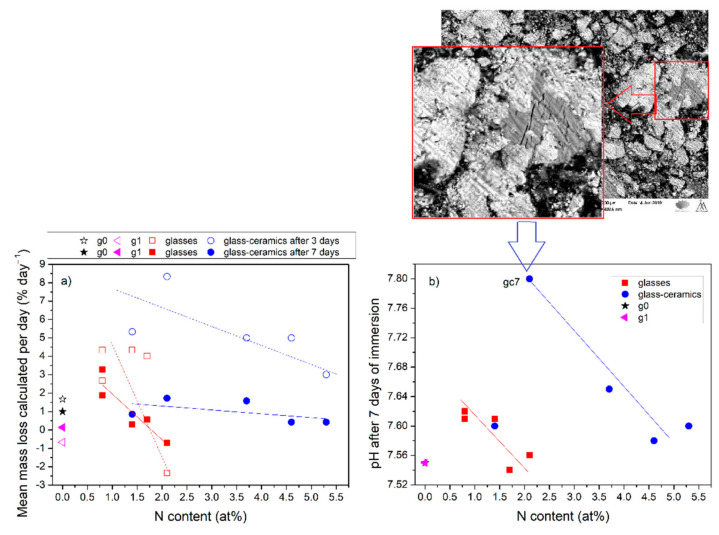
(**a**) Mean % mass loss calculated per day and (**b**) pH values after 7 days of immersion as a function of nitrogen content for glasses and glass-ceramics. SEM micrographs show the enlarged phosphates structures observed for sample gc7.

The N influence on the HAp layer formation in SBF was studied for silicate oxynitride glasses [46] and it was found that varying the N content may allow the level of bioactivity of these glasses to be tailored. Moreover, the initial cytotoxicity tests provided for bioactive silicate-based oxynitride glasses confirmed their non-toxic nature. It was shown that the introduction of nitrogen has no negative effect on the biocompatibility of the silicate bioactive glasses [46]. However, there is no similar research for the phosphate glasses. Therefore, in this work, the influence of N on the dissolution of phosphate-based oxynitride glasses was studied. Figure 6a,b represent the effect of N content on mean mass loss calculated per day of oxynitride samples after immersion in SBF for 3 and 7 days and the pH changes of SBF after immersion of samples for 7 days, respectively. The results were divided into two groups; the first for oxynitride glasses and the second for oxynitride glass-ceramics. For glass-ceramics, the mean % mass loss per day was higher during the first 3 days of immersion and decreased in a long-term test (7 days). This correlation was not observed for oxynitride glasses. However, in both cases, the increase in nitrogen content decreased the mean % mass loss per day. The influence of N incorporation on dissolution is higher in amorphous samples most probably due to the even distribution of nitrogen in glass structure. The correlation with the nitrogen content was also observed for the pH changes of SBF after 7 days of immersion (Figure 6b). The increase in pH of SBF was found to be lower for samples containing higher N amount. The highest mean % mass loss per day and increase in pH was found for glass-ceramic gc7, which was doped with the highest content of Si and rather a small content of N. The slowest mean % mass loss per day was observed for gc6 with the highest content of N (3.2 wt%). These results suggest that N incorporation improved the chemical durability of phosphate glasses through increased network connectivity [24,37,47,48,49] and at the same time decreased their dissolution.

**Figure 7:**
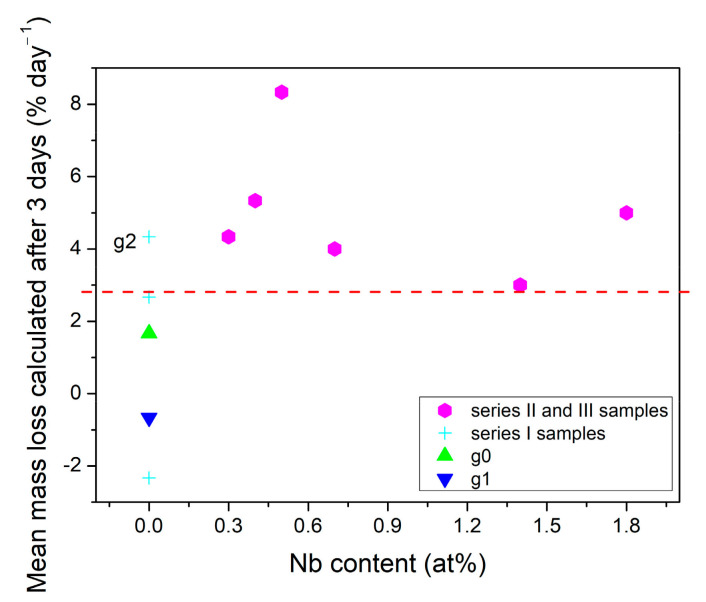
The effect of niobate addition on mean % mass loss calculated per day, during the early steps of the reaction (first 3 days of immersion).

For the silicate glasses, it was found that incorporation of a small content of Nb_2_O_5_ (up to 1.3 mol%), increased the pH during the early steps of the reaction [49]. Similarly, for the phosphate glasses, Nb_2_O_5_ addition improves bioactive properties [50] and release from glass during immersion in SBF [27]. To study the influence of niobate addition on the early step of dissolution properties, the mass loss of the samples was studied as a function of niobate content. Figure 7 shows the comparison of mean % mass loss calculated per day for samples containing niobate (series II and III samples) and glasses lack in niobate (series I samples) for initial stage of immersion (3 days) in SBF. According to the obtained results, niobate-containing samples showed higher mean % mass loss per day during the early steps of dissolution than most of niobate-free glasses (expect g2, marked in Figure 7). However, no correlation was found between the pH changes and niobate addition in all tested samples. Probably, this is due to the presence of crystalline phase in most niobate-containing samples. In [27] it was shown that niobate stayed highly bonded in phosphate glass-ceramic and does not release during immersion in static SBF. Therefore, the effect of the partial crystallization on the dissolution process is also discussed. The mean % mass loss per day was found to be slower for amorphous glasses than for glass-ceramics during the first 3 days of immersion (Figure 6a). Sample gc7, which showed the highest mean mass loss per day contains the cauliflower-like structures made of crystalline phosphates (as shown in SEM pictures in red frame in Figure 6). Therefore, we expect that the presence of sodium and calcium phosphates crystallites support the dissolution process at the beginning step (first 3 days). However, after a longer immersion time (7 days) the mean % mass loss per day of glasses and glass-ceramics became more similar. The influence of heat treatment on long-term biodegradation properties was studied by Maeda et al. [27] for 60CaO-30P_2_O_5_-10Nb_2_O_5_ glasses. They concluded that the crystallization of the glass has improved its durability as a large number of phosphate species formed the calcium pyrophosphates in the glass after the heat treatment. Consequently, only the residual phosphate species in the glassy phase of the glass-ceramic would be released in SBF [27]. In our case, the crystallization process occurred spontaneously during the cooling process. The created crystallites were also made mostly of different sodium and calcium phosphates. However, there is no direct correlation between the content of crystallites (the highest in gc10 and gc11) and the long-term mean % mass loss calculated per day. The reason is that the static dissolution results are an overlap effect of N and niobate incorporation as well as the presence of crystalline phase.

## 5. Conclusions

The in vitro dissolution of phosphate-based oxynitride glasses and glass-ceramics was investigated in SBF under static conditions up to 7 days. In all series, the release of Ca^2+^ from SBF was observed after immersion in the solution, indicating the HAp precipitation on samples’ surfaces. The highest ions (Ca^2+^, Mg^2+^) release from SBF and ions (P^5+^, Si^4+^) release from samples’ surfaces into SBF was observed at the initial stage of immersion (first day). The N incorporation into glass phosphate structure decreased its mean % mass loss at the early step of degradation, but improved its chemical durability through increased network connectivity. Furthermore, the incorporation of N remarkably affected in vitro dissolution of glasses compared to glass-ceramics. The results showed that the niobate incorporation increased the mean % mass loss of bioactive glasses and glass-ceramics. Moreover, no correlation was found between the pH changes and niobate contents. The calcium and sodium phosphates crystallites advanced the beginning of in vitro dissolution in glass-ceramics.

## Figures and Tables

**Table 1:** EDX chemical composition (at%) of samples checked on their fresh fractures. The label g before the sample number referred glass, while gc referred glass-ceramic material. The analysis accuracy is ±5% of value [24].

Element											
ID	Na	K	Ca	Mg	Al	Si	P	Nb	O	N	Ca/P
Matrix glass											
g0	18.1	0.4	5	-	0.3	-	16.8	-	59.5	-	0.30
Reference glass											
g1	17.8	0.4	4.5	-	0.2	0.4	17.3	-	59.4	-	0.26
Series I xSi_3_N_4_-(100-x)(40Na_2_O-20CaO-40P_2_O_5_)											
g2	18.3	0.3	4.5	-	0.3	0.2	17.5	-	58.1	0.8	0.26
g3	17.6	0.2	4.5	-	0.2	0.9	17.4	-	58.5	0.8	0.26
g4	17.1	0.2	4.6	-	0.2	1.3	16.6	-	58	2.1	0.28
Series II xSi_3_N_4_-1Mg-(99-x)(40Na_2_O-20CaO-40P_2_O_5_)											
g5	17.3	1.7	4.5	0.3	0.2	0.5	14.6	0.7	58.7	1.7	0.31
gc6	19.6	3.1	5.4	0.4	0.1	1.8	18.2	1.4	44.7	5.3	0.30
gc7	16.9	1.4	4.6	0.4	0.2	2	15.4	0.5	56.7	2.1	0.30
Series III xMg-(100-x)[1Si_3_N_4_-99(40Na_2_O-20CaO-40P_2_O_5_)]											
g8	20.5	0.5	4.7	0.7	0.2	0.8	16	0.3	54.9	1.4	0.29
gc9	19.6	0.4	4.6	0.9	0.2	0.8	16.3	0.4	55.4	1.4	0.28
gc10	13.9	4.2	8.3	1.3	3.7	1.6	17.6	1.8	43.1	4.6	0.24
gc11	14.4	4.7	8.1	1.6	4.3	1.1	17.2	1.8	42.9	3.7	0.27

**Table 2:** Mass loss % and mean % mass loss calculated per day after initial stage of immersion (3 days) and long-term immersion (7 days) in SBF. Minus sign indicates an increase in mass.

Step of Immersion	Immersion Time (days)	Matrix Glass	Reference Glass	Series I Samples			Series II Samples			Series III Samples			
		g0	g1	g2	g3	g4	g5	gc6	gc7	g8	gc9	gc10	gc11
Mass loss after immersion in SBF (%)													
Initial stage	3	5	−2	13	8	−7	12	9	25	13	16	15	15
Long term	7	7	1	13	23	−5	4	3	12	2	6	3	11
Mean mass loss calculated per day (% day^−1^)													
Initial stage	3	1.67	−0.67	4.33	2.67	−2.33	4	3	8.33	4.33	5.33	5	5
Long term	7	1.40	0.14	1.86	3.29	−0.71	0.57	0.42	1.71	0.28	0.86	0.43	1.57

## Data Availability

The data that support the findings of this study are available from the corresponding author upon reasonable request.

## Supplementary Materials

The following are available online at https://www.mdpi.com/article/10.3390/ma14237425/s1, Figure S1: XRD patterns for glass-ceramic samples from series II and series III. Patterns have been offset by maximum value to allow comparison. Taken from ref. [24], Figure S2. SEM pictures of reference glasses after immersion in SBF for 3 and 7 days, Figure S3. SEM pictures of series I samples glasses after immersion in SBF for 3 and 7 days, Figure S4. SEM pictures of series II samples glasses after immersion in SBF for 3 and 7 days, Figure S5. SEM pictures of series III samples glasses after immersion in SBF for 3 and 7 days, Figure S6. Raw data of SEM and EDX analysis for exemplar sample gc9 after 7 days of immersion in SBF, Table S1. Chemical composition of samples surfaces before and after 3, and 7 days of biodegradation test, measured by EDAX. The possible error is ^+^/_−_ 5% of value.
