# Supplementary material for: In Vitro Dissolution of Na-Ca-P-Oxynitrides
Source: Materials (Basel). 2021 Dec 3;14(23):7425. doi: 10.3390/ma14237425 (PMC8658854; doi:10.3390/ma14237425)
Supplement: Supplementary file 1 [file materials-14-07425-s001.zip › materials-1489545-supplementary.pdf]

materials

MDPI

Article

In Vitro Dissolution of Na-Ca-P-Oxynitrides

Natalia Anna Wójcik [1,2,*], Polina Sinitsyna [3], Sharafat Ali [2], Leena Hupa [3] and Bo Jonson [2]

[1] Advanced Materials Center, Institute of Nanotechnology and Materials Engineering, Gdańsk University of Technology, 11/12 G. Narutowicza Street, 80-233 Gdańsk, Poland
[2] Department of Built Environment and Energy Technology, Linnaeus University, 35195 Växjö, Sweden; sharafat.ali@lnu.se (S.A.); bo.jonson@lnu.se (B.J.)
[3] Johan Gadolin Process Chemistry Centre, Åbo Akademi University, Piispankatu 8, 20500 Turku, Finland; polina.sinitsyna@abo.fi (P.S.); leena.hupa@abo.fi (L.H.)
* Correspondence: natalia.wojcik@pg.edu.pl; Tel.: +48-58348-6606

Abstract: Sodium-calcium-phosphate based oxynitride glasses and glass-ceramics doped with Mg, Si, and Nb were studied in vitro in simulated body fluid (SBF) under static conditions. The release of ions and pH changes up to 7 days of immersion were investigated. The nitrogen incorporation into phosphate glass matrix was found to notably influence in vitro dissolution only of homogenous glasses. Increasing the nitrogen content in the samples decreased the mean mass loss, while the niobate incorporation increased it. The correlation between the nitrogen content and increase in pH of SBF was also observed. The presence of phosphates crystallites was found to support the dissolution process at the beginning step (up to 3 days).

Keywords: bioactive glass-ceramics; simulated body fluid; oxynitride glass-ceramic; in vitro dissolution

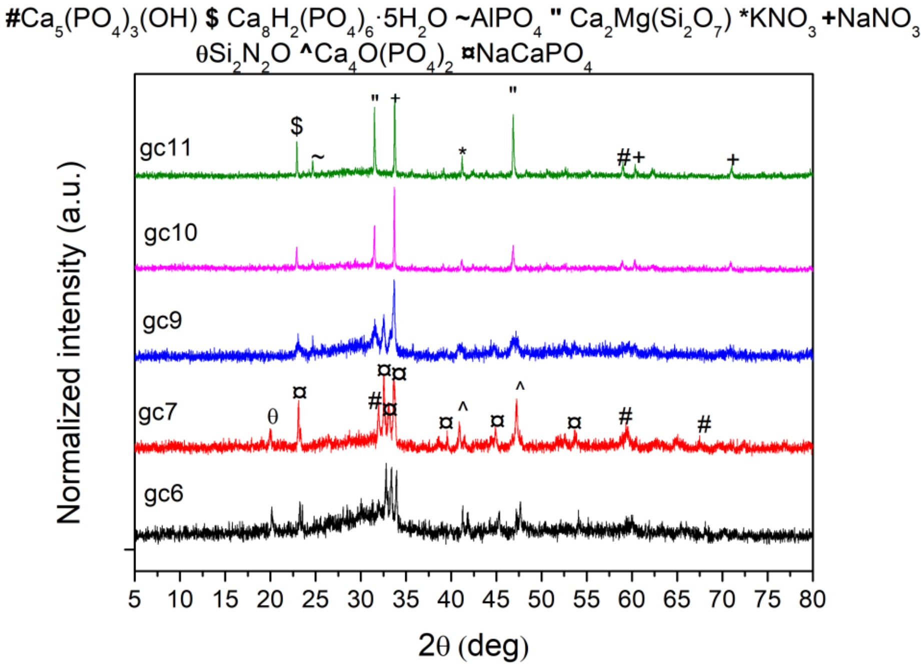

Figure S1. XRD patterns for glass-ceramic samples from series II and series III. Patterns have been offset by maximum value to allow comparison. Taken from ref. [24].

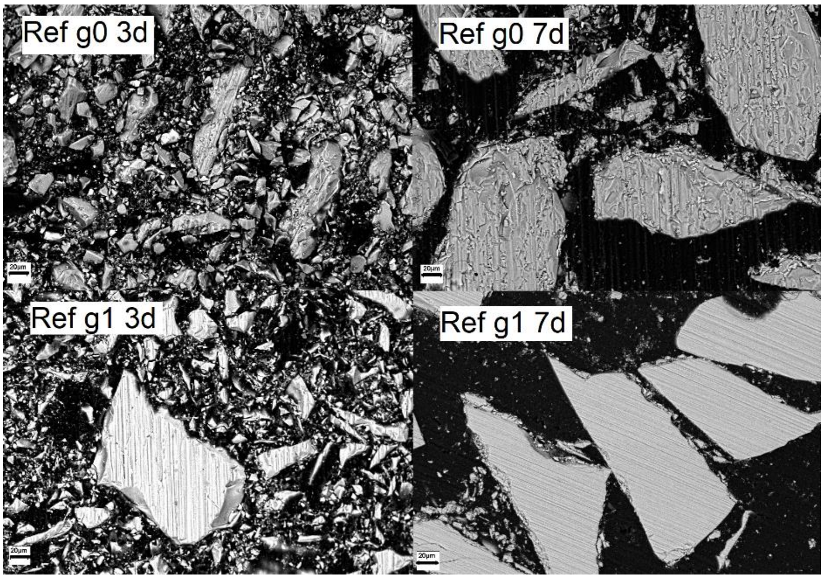

Figure S2. SEM pictures of reference glasses after immersion in SBF for 3 and 7 days.

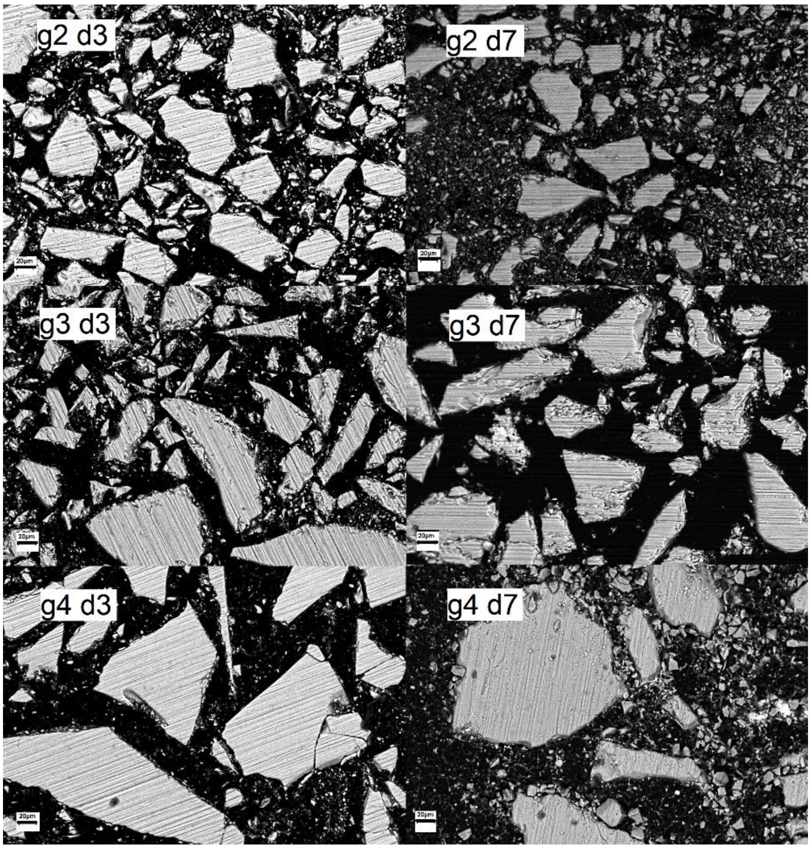

Figure S3. SEM pictures of series I samples glasses after immersion in SBF for 3 and 7 days.

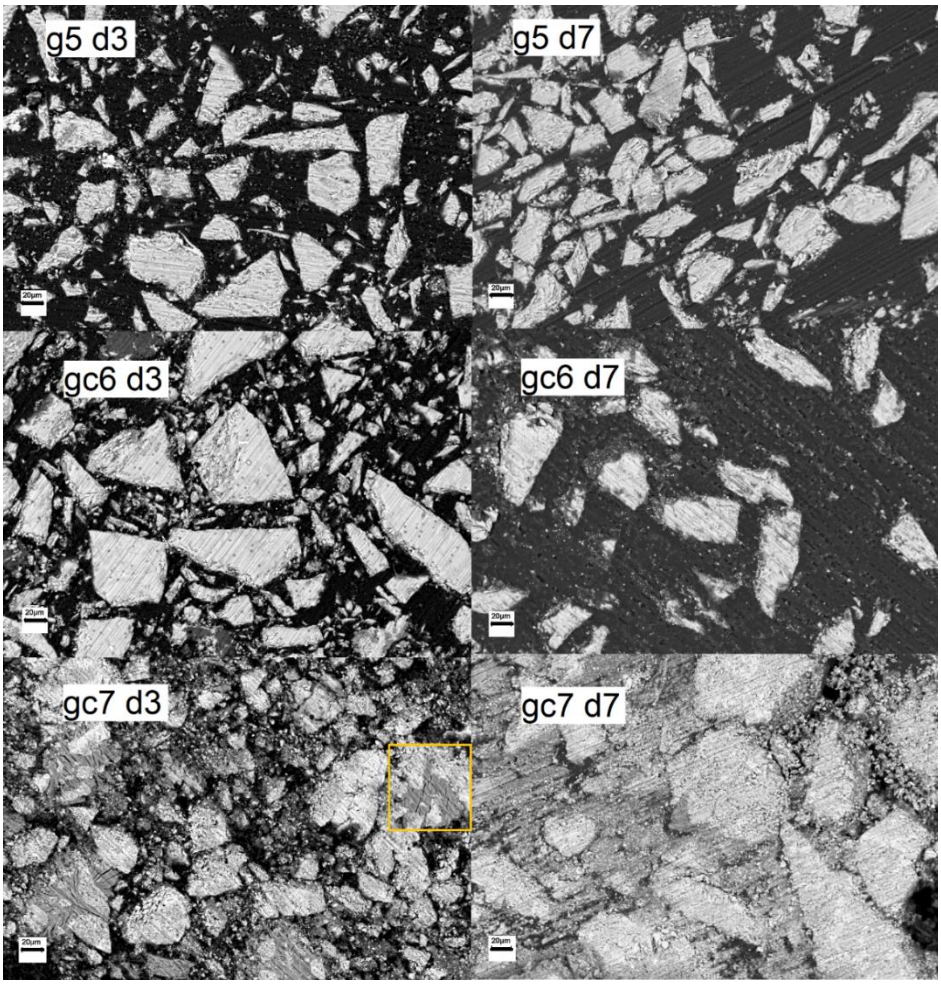

Figure S4. SEM pictures of series II samples glasses after immersion in SBF for 3 and 7 days.

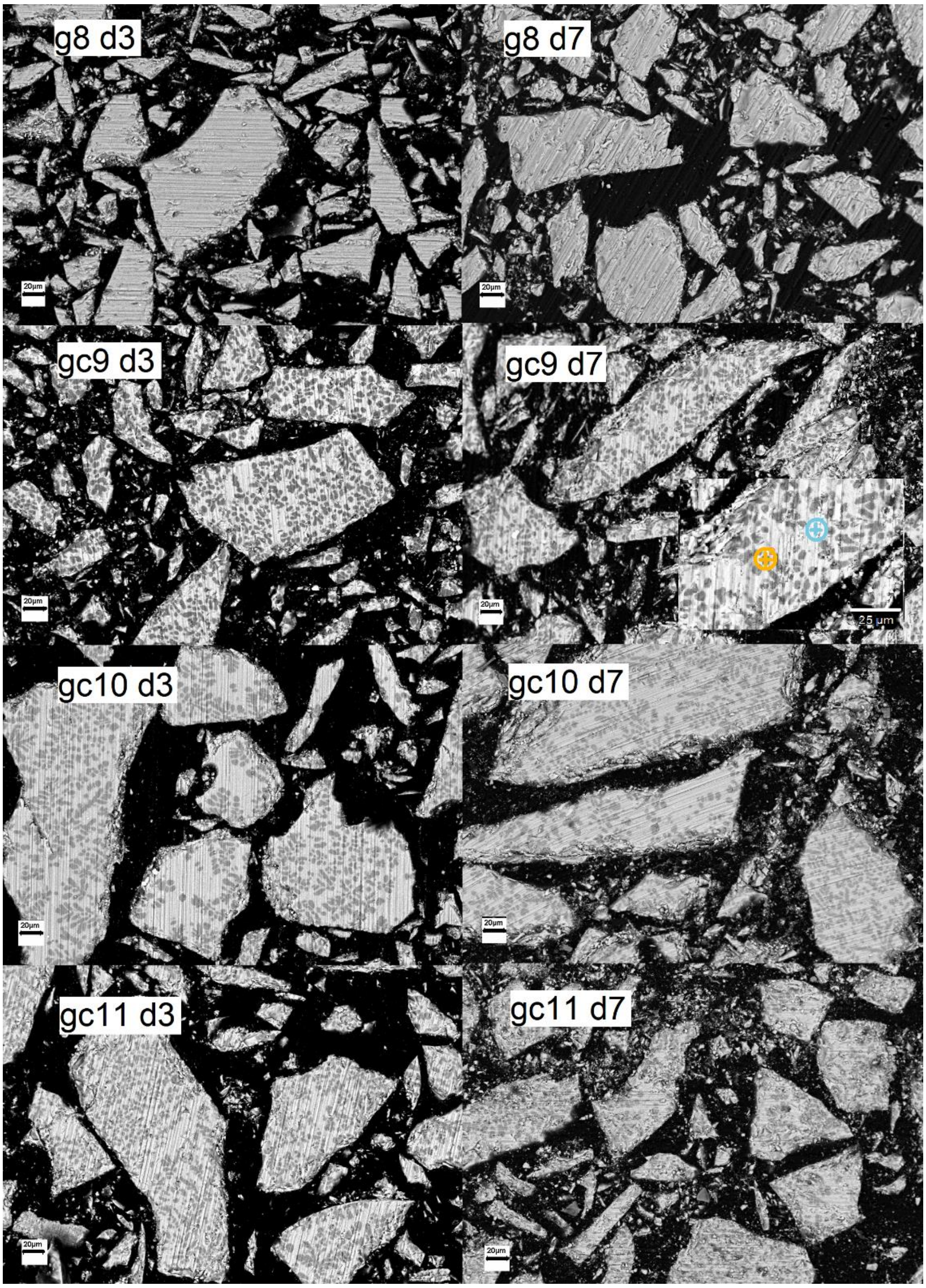

Figure S5. SEM pictures of series III samples glasses after immersion in SBF for 3 and 7 days.

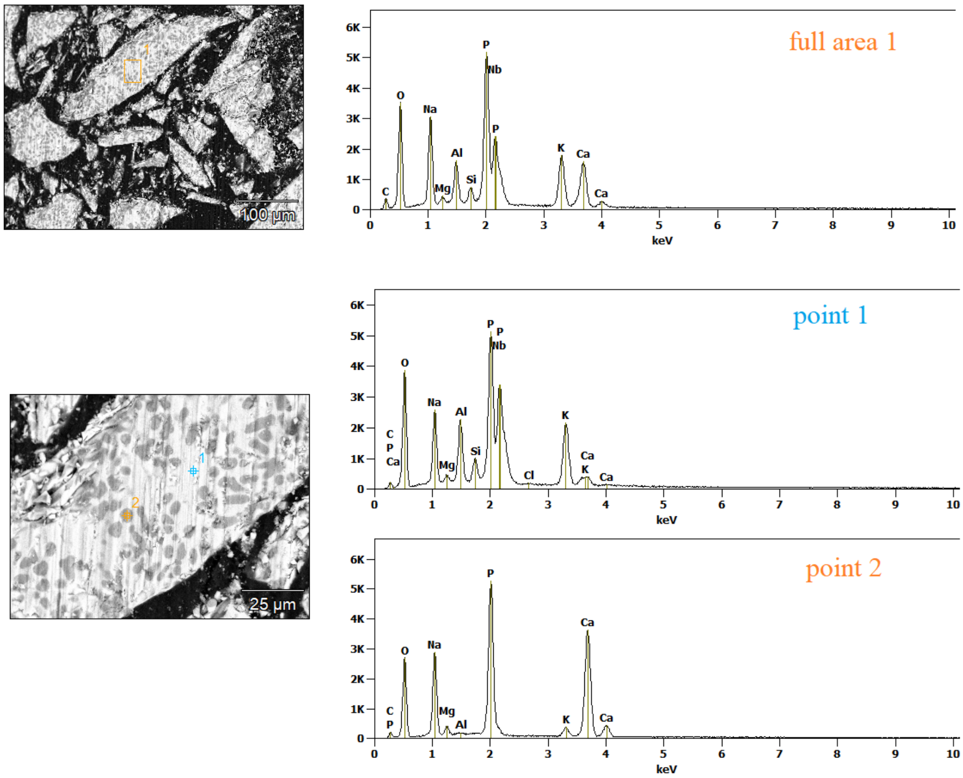

Figure S6. Raw data of SEM and EDX analysis for exemplar sample gc9 after 7 days of immersion in SBF.

Table S1. Chemical composition of samples surfaces before and after 3, and 7 days of biodegradation test, measured by EDAX. The possible error is +/- 5% of value.

ID	Time of immersion on SBF	Na	K	Ca	Mg	Si	P	Nb	Al	Ca/P
	(days)	(wt%)	(wt%)	(wt%)	(wt%)	(wt%)	(wt%)	(wt%)	(wt%)	(at%)
Target glass										
g0	0	19.7	0.7	9.4	-	-	24.6	-	0.4	0.3
	3	19.9	0.4	9.3	-	-	25.5	-	-	0.28
	7	20.2	0.2	8.7	-	-	24.9	-	-	0.27
Reference glass g1										
g1	0	19.4	0.7	8.5	-	0.5	25.3	-	0.3	0.26
	3	16.7	1.6	8.3	-	0.4	22.4	-	6.2	0.29
	7	16.9	1.7	7.5	-	0.4	21.2	-	8.2	0.27
Series I glasses										
g2	0	19.9	0.6	8.6	-	0.3	25.7	-	0.4	0.26
	7	16.8	0.3	5.9	-	1.9	19.3	-	10.7	0.24
g3	0	19.2	0.4	8.5	-	1.2	25.5	-	0.3	0.26
	3	14	5.9	8	-	0.8	21.9	-	5.8	0.28
	7	13.9	6.4	8.2	-	0.8	22.1	-	5.2	0.28
g4	0	18.8	0.3	8.7	-	1.7	24.5	-	0.3	0.28
	7	16.7	0.2	5.5	-	1.9	18.4	-	12.3	0.23
Series II samples										
g5	0	18.5	3	8.3	0.3	0.7	21.1	2.9	0.2	0.31
	7	16.9	5.5	8.7	0.2	0.4	22.5	4	-	0.33
gc6	0	19.3	5.2	9.2	0.4	2.2	24.2	5.7	0.1	0.3
	3	17.3	4	9.2	0.2	2.4	19.8	8	-	0.36
	7	16.3	4.5	8.9	0.1	2.4	19.2	6.6	0.1	0.35
gc7	0	20.8	3	8.6	0.4	2.6	22.4	2.1	0.2	0.3

	3	16.9	3.7	10	0.2	0.9	19.4	7.4	0.1	0.39
	7	17.4	2.3	14.9	0.2	0.7	17.6	6.4	0.1	0.66
Series III samples										
g8	0	22	0.9	8.8	0.8	1	23.2	1.3	0.3	0.29
	3	13.7	8.1	7.7	0.4	1	21	2.9	3.2	0.28
	7	12.1	8.7	7.3	0.3	1.1	21.3	3.2	3.6	0.26
gc9	0	20.9	0.8	8.6	1	1	23.5	1.8	0.2	0.28
	3	12.1	6.4	10.3	0.5	1	15.6	12.5	2.8	0.5
	7	13.2	7	7.2	0.4	1.1	14.4	14.9	3.3	0.39
gc10	0	13	6.7	13.5	1.3	1.8	22.2	6.7	4.1	0.47
	3	12.5	7	10.1	0.7	1.1	17.3	7.9	3.4	0.47
	7	13.7	7.2	7.4	0.8	1.2	16.9	8.7	4.1	0.34
gc11	0	13.4	7.5	13.2	1.6	1.3	21.6	6.8	4.7	0.47
	3	13.3	7	8.9	1.1	1.1	17.2	7.8	3.5	0.4
	7	12.3	7.3	9.1	1	1	17.2	8.5	3.5	0.4
